# Plants as a realized niche for *Listeria monocytogenes*

**DOI:** 10.1002/mbo3.1255

**Published:** 2021-12-17

**Authors:** Hoai‐Nam Truong, Dominique Garmyn, Laurent Gal, Carine Fournier, Yann Sevellec, Sylvain Jeandroz, Pascal Piveteau

**Affiliations:** ^1^ Agroécologie, AgroSup Dijon, CNRS, INRAE University Bourgogne Franche‐Comté Dijon France; ^2^ French Agency for Food, Environmental and Occupational Health & Safety (ANSES), Laboratory for Food Safety, Salmonella and Listeria Unit Paris‐Est University Maisons‐Alfort Cedex France; ^3^ INRAE UR OPAALE Rennes France

**Keywords:** foodborne pathogen, habitat, *Listeria monocytogenes*, microbe‐associated molecular pattern, plant immunity, plant–microbe interaction

## Abstract

*Listeria monocytogenes* is a human pathogen. It is the causative agent of listeriosis, the leading cause of bacterial‐linked foodborne mortality in Europe and elsewhere. Outbreaks of listeriosis have been associated with the consumption of fresh produce including vegetables and fruits. In this review we summarize current data providing direct or indirect evidence that plants can serve as habitat for *L. monocytogenes*, enabling this human pathogen to survive and grow. The current knowledge of the mechanisms involved in the interaction of this bacterium with plants is addressed, and whether this foodborne pathogen elicits an immune response in plants is discussed.

## Graphical abstract

In this review, we summarize current data providing direct or indirect evidence that plants can serve as habitats for *Listeria monocytogenes*, the causative agent of the food‐borne life‐threatening disease listeriosis. The mechanisms involved in the complex interplay between plants and this bacterium are addressed, and whether this pathogen elicits an immune response in plants is discussed. The combination of plant intrinsic factors, extrinsic biotic factors, and abiotic environmental factors draws the boundaries of plant habitat colonization by *L. monocytogenes*.

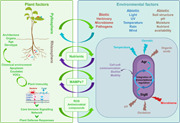

## 1 INTRODUCTION

Understanding the ecology of pathogenic microorganisms requires a thorough knowledge of their habitats and their routes of transmission. *Listeria monocytogenes* (Lm) is a foodborne pathogen that is the causative agent of listeriosis, a serious foodborne disease that affects primarily at‐risk people (pregnant women, elderly, immunocompromised individuals) after consumption of contaminated food. High intraspecific diversity is observed and the species is structured in well‐defined genetic lineages and clonal complexes. Plants interact with microorganisms in their close vicinity and can offer habitats for commensal and human pathogens. Indeed, listeriosis outbreaks have been traced back to preharvest contamination of fresh produce due to the presence of Lm in the farm environment. In that sense, plants must be considered as habitats that are potentially colonized by the human pathogen, and as possible vectors of contamination. To colonize plants bacteria must be able: (i) to utilize available nutrients, (ii) to sense the plant and develop a chemotactic response; (iii) to outcompete other microorganisms and occupy available microniches. In addition, for successful colonization of the rhizoplane or root tissue, microbes must be able to attach to the surface and/or enter root tissue while evading immune responses.

In this review, we discuss the current reports on the occurrence of Lm on plants and the experimental evidence that demonstrates the ability of Lm to colonize plants. We then address the current understanding of the intrinsic and extrinsic factors that underlie plant colonization. Finally, we discuss the current understanding of the contribution of plant biology in providing habitats for Lm and on the interplay between the plant and the human pathogen in light of plant immunity.

## 2 PLANTS OFFER SUITABLE HABITATS FOR THE PERSISTENCE OF *LISTERIA MONOCYTOGENES*

### 2.1 Preharvest contamination and prevalence in market vegetables and fresh produce

Plants offer an environment in which a wide variety of microorganisms can develop including bacteria, fungi, archaea, viruses, and algae. These microorganisms dwell either in the soil close to plants (in the rhizosphere), as epiphytes at the surface of plant organs, or as endophytes within the plants (Fitzpatrick et al., 2020; Leveau, 2019; Pascale et al., 2020). The phyllosphere is composed of the aerial parts of the plant while the rhizosphere is composed of the roots and the surrounding zone of soil under their influence.

Leaves are generally described as oligotrophic and are a hostile environment because of direct solar radiation, large temperature, and humidity fluctuations (Hirano & Upper, 2000; Kadivar & Stapleton, 2003; Leveau, 2019; Redford & Fierer, 2009). Though leaves globally offer harsh environmental conditions, water and nutrients can accumulate locally in epidermal grooves, at the vicinity of glandular trichomes (Schlechter et al., 2019). Natural openings (stomata, hydathodes, etc.) or cracks and wounds at the plant surface are zones where microorganisms can potentially gain access to plants (Chaudhry et al., 2021).

**Figure 1:**
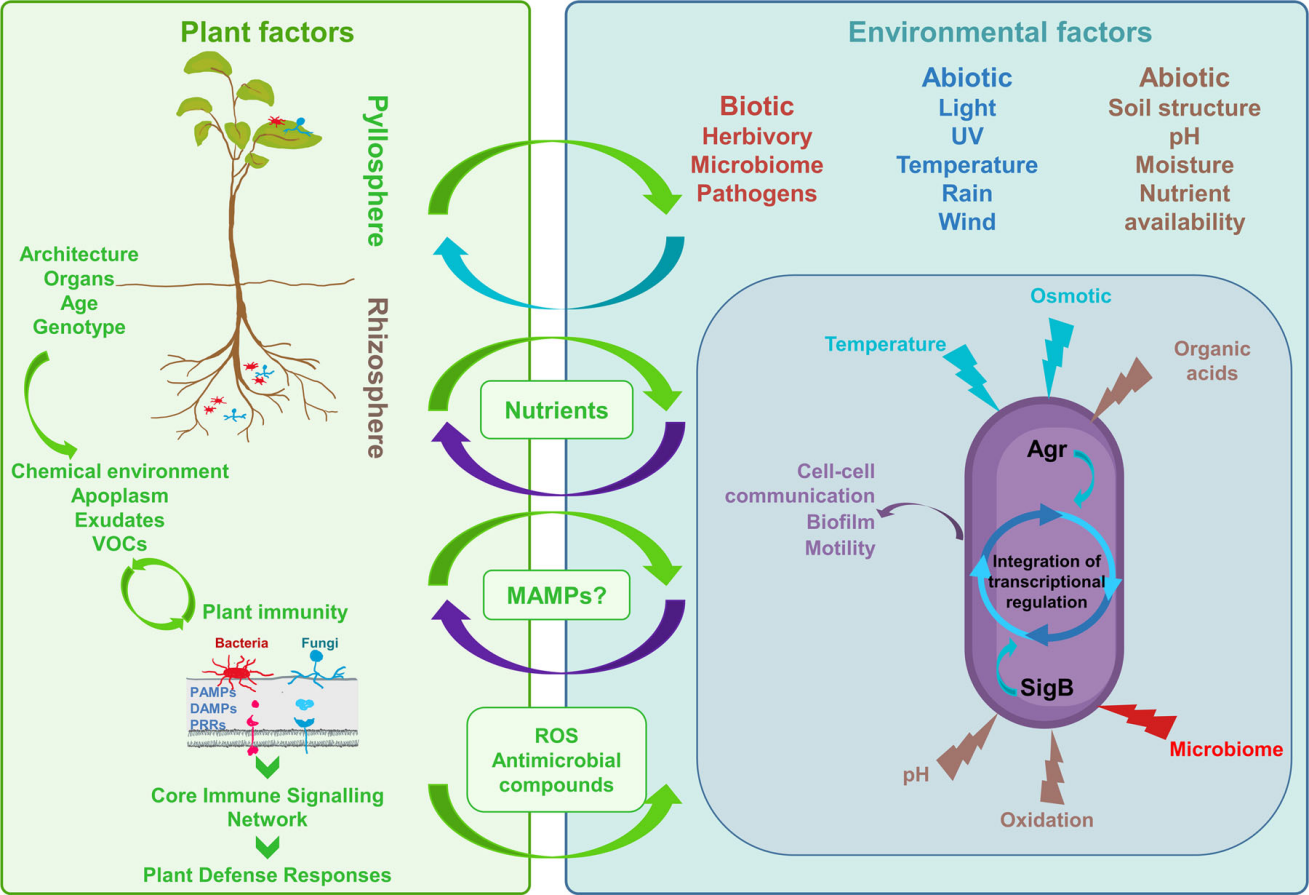
The complex interplay between plants and *Listeria monocytogenes* (Lm). The combination of plant intrinsic factors, extrinsic biotic factors, and abiotic environmental factors draws the boundaries of habitat colonization by Lm

In contrast to the highly fluctuating environment of leaves, soil offers somewhat constant environmental conditions. This complex matrix is composed of a mineral fraction, organic matter, a liquid phase, and a gas phase. Soil is the habitat of complex networks of living organisms from bacteria, Archaea, fungi, viruses, protozoa, nematodes, microarthropods, earthworms, insects, and insect larvae (Briones, 2018; Bunemann et al., 2018; Rabot et al., 2018). Field studies show that Lm can be found in soil but detection is generally uneven according to complex combinations of edaphic, landscape, and meteorological factors (Chapin et al., 2014; Strawn, Fortes, et al., 2013; Strawn, Grohn, et al., 2013; Weller et al., 2015). The overall conclusion of these studies is the multifactorial dimension of the prevalence and fate of Lm in soil. For example, soil pH, cation exchange capacity, water holding capacity, mineral composition, and temperature are important abiotic factors (Locatelli et al., 2013; Mclaughlin et al., 2011; Sidorenko et al., 2006). Moreover, soil microbial diversity and community structure are key factors controlling the fate of Lm in soil (Spor et al., 2020; Vivant et al., 2013). As a consequence, preharvest contamination also depends on environmental biotic and abiotic factors as well as agronomic practices (Miceli & Settanni, 2019). Within the soil, roots can harbor Lm. Indeed, exudation of up to 20% of the carbon fixed by plants and 15% of their nitrogen at the root/soil interface makes the rhizosphere a nutrient‐rich habitat (Haichar et al., 2016; Venturi & Keel, 2016). Root tips, root hairs, cracks at the emergence of lateral roots, and wounds are zones vulnerable to microbial entry (Mercado‐Blanco & Prieto, 2012). These plant habitats are shaped by intrinsic factors of the plant (i.e., plant phenotype, genotype, age, physiology), abiotic factors (climate, soil properties, nutrient availability, etc.), and biotic factors (commensal/beneficial microorganisms and pathogen pressure) (Figure 1).

Direct and indirect evidence confirm that plants are suitable habitats for Lm. *Listeria* spp. and Lm can be isolated from fresh produce farms (Bilung et al., 2018; Chapin et al., 2014; Prazak et al., 2002; Szymczak et al., 2014; Weller et al., 2015). Preharvest contamination by Lm has been reported for several kinds of fresh produce including strawberries (prevalence 10%), potatoes (prevalence 15%), and parsley (prevalence 5%) but the contamination depended on the fertilization strategy (organic or chemical fertilizers) (Szymczak et al., 2014). This suggested that Lm presence in fruit or vegetables could in part be due to contamination from the organic fertilizer derived from animal feces. Others reported contamination of cabbages (Prazak et al., 2002), carrots (Kljujev et al., 2018), spinach (Weller et al., 2015), and other leafy greens (basil, dill, garden cress, kales, lettuce, mint, parsley, purslane, rockets) (Aytac et al., 2010). These reports confirm the preharvest transfer of Lm to growing plants.

Furthermore, the occurrence of contaminated raw vegetables and fresh produce at retail has been reported from several countries (Table 1). Lm prevalence on vegetables, herbs, and mushrooms is variable among countries. Although contamination may occur anywhere along the food chain and depends on many factors (Alegbeleye et al., 2018; K. Honjoh et al., 2018; Miceli & Settanni, 2019; Smith et al., 2018), these data give indications on the type of fresh produce and vegetables potentially contaminated in the field.

**Table 1:** Occurrence of contaminated raw vegetables and fresh produce at retail

Country	Vegetable type	Level of contamination (%)	References
Estonia	Fruits and vegetables	Up to 3	Kramarenko et al. (2013)
Soudan	Prevalence on cucumber, cabbage, carrot, tomato, and lettuce	0.41–5	Ajayeoba et al. (2016)
South Africa	Cabbage and spinach	7	Du Plessis et al. (2017)
India	Tomatoes	11	
	Coriander leaves	50	Pingulkar et al. (2001)
	Spinach	25	
	Cabbage	25	
	Brinjal, cauliflower, Chappan Kaddu, chili	20	Soni et al. (2014)
	Dolichos bean and tomato	10	
Malaysia	Carrots	24.2	
	Sweet potatoes	28.1	
	Indian pennyworts	25	
	Japanese parsley	39.4	Ponniah et al. (2010)
	Winged beans	34.4	
	Yardlong beans	40.6	
	Tomatoes	21.9	
	cucumbers	43.8	
Brazil	Leafy greens and vegetables	1.2	De Oliveira et al. (2010)
South Korea	Fresh fruits and vegetables	0–1.7	Seo et al. (2010), Tango et al. (2018)
Japan	Leaves, roots, bulbs, mushrooms, and sprouts	0	Inoue et al. (2000)
	Iceberg lettuce	0	Koseki, Mizuno, Kawasaki, et al. (2011)
China	Vegetables	1.7	Yu and Jiang (2014)
	Vegetables and herbs	2.8	Chen et al. (2015)
	Vegetables	5.7	Wu et al. (2015)
	Vegetables	2	Wang et al. (2017)
	Vegetables and herbs	7.8	Chen et al. (2019)
	Mushrooms	21.2–31.5	Chen et al. (2015, 2018), Wu et al. (2015)
Ireland	Mushrooms	3.8	Pennone et al. (2018)

Overall, contamination is generally low. Indeed, based on prevalence data available in the literature, mathematical modeling suggested that the probability of contamination of unprocessed fresh vegetables with more than 10 Lm/g was 1.44% and it dropped to 0.17% for rates of contamination over 1000 Lm/g (Crepet et al., 2007).

### 2.2 Experimental data on in vitro plant colonization

**Figure 2:**
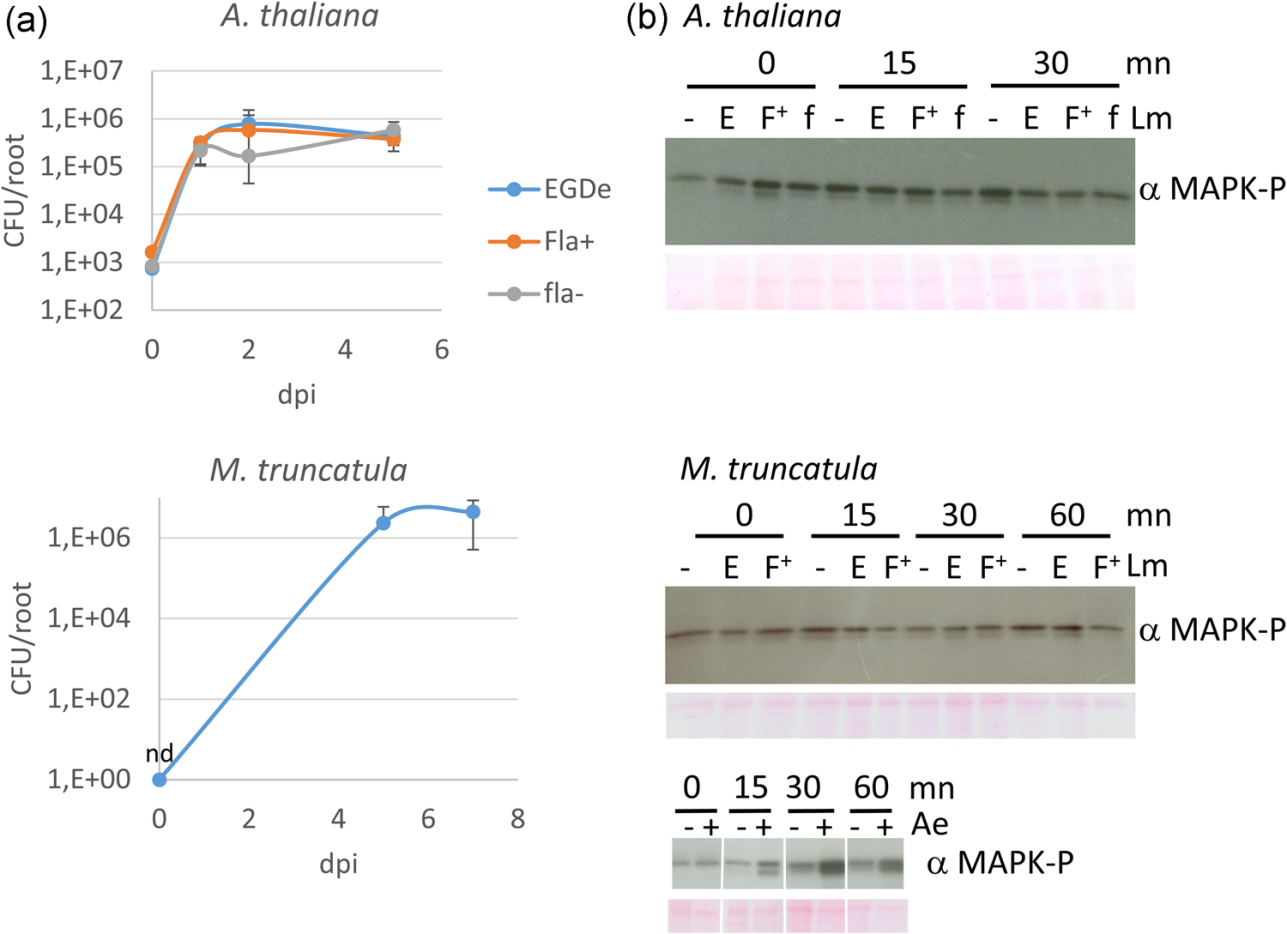
Experimental evidence of the growth of *Listeria monocytogenes* (Lm) on roots of the model plants *Arabidopsis thaliana* and *Medicago truncatula* (a) and absence of MAPK activation after inoculation of roots with Lm (b). (a) Roots were inoculated with Lm (10^4^ CFU/root for *A. thaliana* and 10^3^ CFU/root for *M. truncatula*). (b) Roots were inoculated with Lm (10^8^ CFU/root) and MAPK activation was assessed at different time points by Western blot analysis using an antibody recognizing the activated form of MAPK (MAPK‐P). dpi, days postinoculation; E, *L. monocytogenes* EGDe parental strain; Fla +/ F^+^, constitutive FlaA producer mutant derived from Lm EGDe; fla−/f, FlaA‐deficient mutant derived from Lm EGDe; MAPPK, mitogen‐activated protein kinase; nd, <20 CFU/root; −, negative control; +, positive control (inoculation of *M. truncatula* with the phytopathogen *Aphanomyces euteiches* [Ae])

Many studies performed under laboratory conditions confirmed that Lm can colonize and persist on plants. Table 2 presents a selection of these studies. Because of food safety issues, many investigations addressed the colonization of edible plants. Lm inoculation at the surface of roots or leaves resulted in population increase and colonization of parsley (*Petroselinum crispum*) (Bardsley et al., 2019; Kljujev et al., 2018), lettuce (*Lactuca sativa*) (Chitarra, Decastelli, et al., 2014; K. Honjoh et al., 2018; Jablasone et al., 2005; Kljujev et al., 2018; Shenoy et al., 2017; Standing et al., 2013), corn salad (*Vallerianella locusta*) (Chitarra, Decastelli, et al., 2014; Hofmann et al., 2014), spinach (*Spinacia oleracea*) (Hofmann et al., 2014; Jablasone et al., 2005; Kljujev et al., 2018), mustard spinach (*Brassica rapa*) (Koseki, Mizuno, Yamamoto, 2011b), cultivated rocket (*Eruca sativa*) (Chitarra, Decastelli, et al., 2014; Settanni et al., 2012), wild rocket (*Diplotaxis tenuifolia*) (Chitarra, Decastelli, et al., 2014), cress (*Lepidium sativum*) (Jablasone et al., 2005), carrot (*Daucus carota*) (Kljujev et al., 2018), radish (*Raphanus raphanistrum*) (Jablasone et al., 2005), sweet pepper (*Capsicum annuum*) (Füstös et al., 2017), basil (*Ocimum basilicum*) (Bardsley et al., 2019; Chitarra, Decastelli, et al., 2014; Settanni et al., 2012), cilantro (*Coriandrum sativum*), dill (*Anethum graveolens*) (Bardsley et al., 2019), tomato (*Solanum lycopersicum*) (K.‐I. Honjoh et al., 2016), cantaloupe (*Cucumis melo*) (Nyarko et al., 2016), peach (*Prunus persica*), plum (*Prunus domestica*) (Collignon & Korsten, 2010), sweet corn (*Zea mays*) (Kljujev et al., 2018), and alfalfa sprouts (*Medicago sativa*) (Adhikari et al., 2019). Nonedible plants can be colonized by Lm. For example, *Arabidopsis thaliana* (Milillo et al., 2008), *Festuca arundinacea* (Marinho et al., 2020), *Cajanus cajan* (Sharma et al., 2020), and *Medicago truncatula* (Figure 2a) can support Lm growth. Experiments in our lab suggest Lm can reach populations of 10^6^–10^7^ CFU/plant following root inoculation of *F*. *arundinacea* and *C. cajan* (Marinho et al., 2020; Sharma et al., 2020) and confocal microscopy observations confirmed that Lm can establish as biofilms (L. Gal et al., personal communication).

**Table 2:** Plant colonization by *Listeria monocytogenes* under laboratory conditions^a^

Plant species	Culture condition inoculation doses	Detection methods	Localization and development	References
Lettuce *L. sativa*	Axenic systems, 10^6^–10^7^ CFU/ml	Enumeration on micrographs at 21 dpi	In the surface layers and inside of root	Kljujev et al. (2018)
	Axenic systems, irrigated with 10^7^ CFU/ml	Surface disinfection followed by enumeration on plates	inside of leaves up to 80 days	Chitarra, Decastelli, et al. (2014)
	Standard or autoclaved potting mix, top soil or in vitro, 10^8^ CFU/ml	Immunocytochemistry with Lm expressing GFP Enumeration on plates, up to 21 dpi	Internalizes in all major tissue types No detection in 75% top soil but detection in vitro	Shenoy et al. (2017)
	Seedlings cultivated on vermiculite and hydroponically, 10^5^ CFU/ml	Surface disinfection followed by enumeration on plates, light, and TE microscopy	Inside roots and leaves	Standing et al. (2013)
	Autoclaved commercial soil, 10^4^–10^8^ CFU/g	Enumeration on plates	Detection at low levelNo internalization into leaf detected	K. Honjoh et al. (2018)
	Germinated on dampened sterile filter paper disks and solidified hydroponic solution, seeds soaked in cells suspension (10^2^ CFU/ml)	Enumeration on plates at 9 and 49 dpi	Growth on germinating seeds observed Detection on surface up to 49 daysNo internalization detected	Jablasone et al. (2005)
Spinach *S. oleracea*	Axenic systems, 10^6^–10^7^ CFU/ml	Enumeration on 3D micrographs at 21 dpi	In the surface layers and inside of root	Kljujev et al. (2018)
	Axenic system, 4 x 10^1^ to 4 x 10^6^ CFU/ml Soil, up to 2.4 x 10^7^ CFU/g for slurry setups and 1.6 x 10^7^ for manure setups	PCR detection at 21 dpi	Inside root and shoot Few samples independent of the spiking doses were tested positive	Hofmann et al. (2014)
	Germinated on dampened sterile filter paper disks and solidified hydroponic solution, seeds soaked in cells suspension (10^2^ CFU/ml)	Enumeration on plates at 9 and 49 dpi	Growth on germinating seeds observed Detection on surface up to 49 days No internalization detected	Jablasone et al. (2005)
Corn salad *V. locusta*	Axenic system, 4 x 10^1^ to 4 x 10^6^ CFU/ml	PCR detection at 21 dpi	Detected at inoculation doses of less than 4 × 10^2^ CFU/ml in root, 4 × 10^3^ CFU/ml in shoot	Hofmann et al. (2014)
	Soil, up to 2.4 × 10^7^ CFU/g for slurry setups and 1.6 × 10^7^ for manure setups	PCR detection at 21 dpi	Few samples independent of the spiking doses positive	Hofmann et al. (2014)
	Axenic systems, irrigated with 10^7^ CFU/ml	Surface disinfection followed by enumeration on plates	Not detected inside of leaves	Chitarra, Decastelli, et al. (2014)
Basil *O. basilicum*.	Greenhouse spray of above‐ground parts at 10^6^ CFU/ml with 3 ml	Enumeration on plates	Detection on surface up to 28 days	Bardsley et al. (2019)
	Axenic systems, irrigated with 10^7^ CFU/ml	Surface disinfection followed by enumeration on plates	No internalization into leaf detected	Chitarra, Decastelli, et al. (2014)
	Soil, 2.5 × 10^8^ CFU/g	Enumeration on plates	Failure to detect transfer from soil	Settanni et al. (2012)

^a^ Data on the ability of Lm to colonize plants under laboratory conditions is available with the following plant species: parsley (*Petroselinum crispum*) (Bardsley et al., 2019; Kljujev et al., 2018), lettuce (*Lactuca sativa*) (Chitarra, Decastelli, et al., 2014; Honjoh et al., 2018; Jablasone et al., 2005; Kljujev et al., 2018; Shenoy et al., 2017; Standing et al., 2013), corn salad (*Vallerianella locusta*) (Chitarra, Decastelli, et al., 2014; Hofmann et al., 2014), spinach (*Spinacia oleracea*) (Hofmann et al., 2014; Jablasone et al., 2005; Kljujev et al., 2018), mustard spinach (*Brassica rapa*) (Koseki, Mizuno, Yamamoto, 2011), cultivated rocket (*Eruca sativa*) (Chitarra, Decastelli, et al., 2014; Settanni et al., 2012), wild rocket (*Diplotaxis tenuifolia*) (Chitarra, Decastelli, et al., 2014), cress (*Lepidium sativum*) (Jablasone et al., 2005), carrot (*Daucus carota*) (Kljujev et al., 2018), radish (*Raphanus raphanistrum*) (Jablasone et al., 2005), sweet pepper (*Capsicum annuum*) (Füstös et al., 2017), basil (*Ocimum basilicum*) (Bardsley et al., 2019; Chitarra, Decastelli, et al., 2014; Settanni et al., 2012), cilantro (*Coriandrum sativum*), dill (*Anethum graveolens*) (Bardsley et al., 2019), tomato (*Solanum lycopersicum*) (Honjoh et al., 2016), cantaloupe (*Cucumis melo*) (Nyarko et al., 2016), peach (*Prunus persica*), plum (*Prunus domestica*) (Collignon & Korsten, 2010), sweet corn (*Zea mays*) (Kljujev et al., 2018), alfalfa sprouts (*Medicago sativa*) (Adhikari et al., 2019), pigeon pea (*Cajanus cajan*) (Sharma et al., 2020), Arabidopsis (*Arabidopsis thaliana*) (Milillo et al., 2008), and Fescue grass (*Festuca arundinacea*) (Marinho et al., 2020; Sharma et al., 2020). The table presents major findings only when more than one paper is available for a given plant species.

As indicated in Table 2, the reports available involved different plant species, experimental systems, and inoculation methods (Table 2). One major limitation of most of them is the use of axenic plants. Because of the absence of other microorganisms, these experiments are best‐case scenarios that demonstrate that most plants can act as a fundamental niche for Lm.

### 2.3 Consumption of herbs, vegetables, or plants may be responsible for foodborne outbreaks (FBO) of listeriosis

In 2019, The European Food Safety Authority published the results of a survey on the incidence of Lm in 2357 ready‐to‐eat (RTE) fruit and vegetable products. The overall incidence was 1.7% (Boelaert et al., 2021).

In recent years, several FBOs of listeriosis have been recorded in Europe and traced back to the consumption of frozen corn (2018; 32 cases, six deaths), frozen vegetables (2018; 53 cases, nine deaths), and Korean imported enoki mushrooms (2020; 36 cases, four deaths). In the United States consumption of contaminated frozen vegetables (2016; nine cases, three deaths), packaged salads (2016; 19 cases, one death), and bean sprouts (2014; five cases, two deaths) resulted in listeriosis cases.

Although contaminated herbs and vegetables can be vectors of listeriosis, source tracking is very difficult. Very limited longitudinal data are currently available (Kljujev et al., 2018; Smith et al., 2018; Q. F. Sun et al., 2021) from preharvest environments to food processing factories and eventually retail. Because of the increasing trend toward minimally processed, healthy foods in industrialized countries, filling this lack of data is critically important to mitigate health hazards linked to the consumption of plants and vegetables contaminated with Lm.

## 3 MECHANISMS UNDERLYING Lm GROWTH, PERSISTENCE, AND SURVIVAL ON PLANTS

Independent of the habitat, colonization and persistence rely on complex interplays between the local conditions of the environment surrounding Lm and its ability to sense and respond to environmental cues in accordance with its intrinsic characteristics (Figure 1). So far, several steps have been described in the course of plant colonization but information on the mechanisms triggered during plant colonization remains scarce. The stochastic, nonspecific adhesion of bacterial cells to plant surfaces is followed by their irreversible attachment, followed by active production of exopolysaccharides, multiplication, colonization of the plant surface, and persistence (Collignon & Korsten, 2010; Kyere et al., 2019).

### 3.1 Lm attachment to plants

The contribution of flagella to attachment and colonization of alfalfa, radish, and broccoli sprouts has been investigated in three genotypes of Lm (Gorski et al., 2009). Colonization was impaired in deletion mutants affected in flagella synthesis but results depended on the type of sprout and the genetic background of Lm strains (Gorski et al., 2009). Thus, the absence of flagellum affects the colonization of some plants but this is strain‐dependent. Among the genes required for the synthesis of the flagellar rotor, disruption of *motAB* had a significant effect on surface attachment to radish tissues. However, deletion of *motAB* did not impact root attachment on sprouts but the fitness of the mutants was significantly lower than the parental strains during co‐inoculation experiments. This suggests that motility improves colonization fitness. Conversely, colonization of cut cabbage was not affected by motility (Palumbo et al., 2005).

The lectin‐mediated attachment mechanism is likely to be active during bacteria–root interactions (Danhorn & Fuqua, 2007; Wheatley & Poole, 2018). Indeed, agglutination assays showed that Lm reacts to different plant lectins in a strain‐specific manner (Facinelli et al., 1998; Slifkin & Doyle, 1990). However, lectins of *Canavalia ensiformis* and *Punica granatum* have antibiofilm activities against Lm and other bacteria (Jin et al., 2019; Silva et al., 2021). This suggests that lectins of some plant species may limit adhesion to their surface.

Xyloglucan and pectins are plant cell wall components that affect Lm attachment (Tan et al., 2015). Moreover, a cellulose‐binding protein enables Lm attachment to lettuce (Bae et al., 2013). Altogether, these reports highlight the importance of the structures and components of plant cell walls in the attachment of Lm.

Information on transcriptome variations triggered by plant colonization is limited, and genes whose expression is required during plant colonization remain to be duly identified. In one study, a differential display approach was undertaken to compare the Lm gene expression profile under two conditions. In the first, Lm was inoculated on cut cabbage. In the second Lm was cultivated in standard laboratory conditions (Palumbo et al., 2005). Although several genes were transcribed differentially, including genes contributing to cell surface characteristics, disruption of some of these genes did not impede attachment and growth on cabbage.

### 3.2 Nutrient utilization during colonization/proliferation of Lm on plants

The growth of Lm on plants relies on its ability to utilize plant‐derived nutrients (Palumbo et al., 2005). Indeed plants release to their environment a blend of compounds produced constitutively or in response to environmental cues, including abiotic and biotic stressors (Bais et al., 2006; Chaudhry et al., 2021; Jacoby et al., 2020; Sasse et al., 2018). The composition of these nutrient‐rich exudates depends on the plant species, age, nutrition, and physiology (Bais et al., 2006). Exudates are mixtures of low molecular weight (organic acids, amino acids, sugars, secondary metabolites) and high molecular weight (mucilage, proteins) C‐rich molecules. Leakage of nutrients at root junction sites, after tissue wounding or phytopathogen infection, can be another source of nutrients available for the development of Lm (Brandl, 2006). The increase in numbers of Lm on seeds germinating on sterile dampened filter papers confirms that Lm can make use of the plant compounds for growth, attaining levels of 5.5–6.9 log CFU/g (Jablasone et al., 2005). Furthermore, when Lm was inoculated on fresh‐cut cabbage, higher transcription of genes associated with transport, carbohydrate metabolism, amino acid, vitamin, and nucleotide biosynthesis suggests that Lm can transport and metabolize a wide range of plant‐derived resources (Palumbo et al., 2005).

Though leaf surfaces are oligotrophic environments, limited amounts of exudates can be released in the phyllosphere. The presence of nitrogen in leaf exudates was a critical factor promoting the growth of human pathogens on lettuce leaves (Brandl & Amundson, 2008), and bacterial multiplication on leaves is supported locally by discrete zones providing higher concentrations of sugars (Leveau & Lindow, 2001). Still, the leaf habitat displays harsher conditions than roots (Koseki, Mizuno, Yamamoto, 2011). For example, microscopic examination of germinated sprouts confirmed that Lm was preferentially localized on root hairs rather than on leaves (Gorski et al., 2004, 2009). However, these studies were performed with axenic sprouts and the absence of other microorganisms is a major bias in comparison to field conditions.

### 3.3 Stress response

Although plants provide habitats for microorganisms, the production of specific molecules can induce stressful conditions for bacteria (Foreman et al., 2003). Coping with harsh conditions is a prerequisite for plant colonization. For example, intrinsic resistance to cumene hydroperoxide in a collection of Lm strains was correlated with higher colonization of sprouts, regardless of the type of sprout used in the study, but the results were to some extent strain‐dependent (Gorski et al., 2008). The authors proposed that resistance to oxidative stress was one of the many factors contributing to the success of root colonization. The general stress response plays indeed a key role in the process of habitat colonization. Sigma B is the essential factor in the response of Lm to stressors (low pH, oxidizing conditions, starvation, and osmotic variations); it coordinates the transcription of approximately 10% of the genome (Ferreira et al., 2001, Fraser et al., 2003). Deletion of the gene encoding Sigma B (*sigB*) did not obliterate growth and survival in commercial potting soil nor on radish but the mutant population was 1–2 orders of magnitude lower than the parental strain (Gorski et al., 2011). These results were confirmed in another genetic background during in vitro root colonization of *F. arundinacea* and survival in agricultural soil microcosms (Marinho et al., 2020). These data suggest that regulation of transcription by Sigma B is required for optimal adaptation and survival in the rhizosphere but not in the initial steps of attachment to root surfaces.

Further root colonization defects were observed with a strain (*∆agrA∆sigB*) with a double mutation that affected both the general stress response and cell to cell communication (Marinho et al., 2020); this suggests that both, cell to cell communication and general stress response contribute to success during root colonization.

A variety of plant secondary metabolites act as defense compounds. Several volatiles produced by plant leaves or roots display antimicrobial properties against Lm (Kawacka et al., 2021). These include benzenoids, phenylpropanoids, phenolics, and terpenoids released by essential oils (Farré‐Armengol et al., 2016). Interference with adherence ability, biofilm formation, and bacterial cell membrane disruption appear to be the mechanisms of action of some of these plant‐derived antimicrobial compounds (Kawacka et al., 2021). As the experiments were generally performed with concentrated extracts or purified compounds, how these data relate to plant/Lm interaction in vivo remains to be assessed.

### 3.4 Biotic interactions with plant microbiome

Whatever the habitat, one of the major extrinsic factors driving the fate of Lm is the presence of other microorganisms. Plants are metaorganisms harboring complex communities of microorganisms collectively referred to as the plant microbiome. The abundance and composition of microbiomes are different on leaves (phyllosphere) and roots (rhizosphere). The rhizospheric microbiome is composed of various classes of microbes: fungi, bacteria and archaea, actinomycetes, protozoa, nematodes (Mendes et al., 2013), and algae (Lee & Ryu, 2021). Bacteria are a major component of the plant microbiome contributing to plant growth, protection from environmental stressors (Devarajan et al., 2021), protection from pathogens (Ritpitakphong et al., 2016) and they are essential to carbon and nitrogen cycles (Abadi et al., 2021; Reed et al., 2010). Phyllosphere microorganisms are mainly bacteria (Alphaproteobacteria, Gammaproteobacteria, and the phyla Bacteroidetes and Actinobacteria). Fungi are also detected in the phyllosphere and appear to be highly diverse (Kembel et al., 2014; Vorholt, 2012). Recent studies suggest that the soil contributes to phyllosphere microbes in addition to parental material and the atmosphere (Grady et al., 2019; Zheng & Lin, 2020; Zhou et al., 2021).

Experiments in unplanted soil microcosms clearly showed that soil microbiomes can act as efficient barriers preventing invasion by Lm (Dowe et al., 1997; Locatelli et al., 2013; Mclaughlin et al., 2011; Moynihan et al., 2015). Although the overall diversity of soil microbiomes plays a key role in generating hostile conditions for Lm, the phylogenetic composition has to be considered as well (Spor et al., 2020; Vivant et al., 2013). Experiments carried out on soil microcosms planted with the *Poaceae F. arundinacea* have demonstrated that the presence of plants improved to some extent the survival of the pathogen (L. Gal et al., personal communication). However, unlike in vitro, no growth could be observed and the population of Lm in the rhizosphere gradually declined. Therefore it is likely that, compared to bare soil, the rhizosphere environment is favorable for the survival and maintenance of Lm. The relationship between the characteristics of plant microbiome and the settlement of Lm in the rhizosphere or leaves has yet to be documented. Similar trends are expected in the rhizosphere as in unplanted soil. For example, specific strains of *Azotobacter chroococcum*, *Bacillus megaterium*, and *Pseudomonas fluorescens* can control Lm in the rhizosphere possibly through a combination of competition and antibiosis (Sharma et al., 2020). In conclusion, the plant microbiome is the major factor limiting Lm niche breadth. In the future, implementing farming practices favoring microbiome diversity is an exciting field of investigation to limit preharvest contamination and improve food safety.

### 3.5 Conflicting information on Lm internalization in plant tissues

Internalization of human pathogens in plant tissues raises further food safety issues. Indeed, internalized bacteria, whether present in the extracellular space or intracellular compartments are protected from removal by washing and surface disinfection, and therefore may threaten consumers' health when fresh produce is eaten raw (Erickson, 2012). Whether or not Lm colonizes plants internally is still a matter of debate and conflicting reports are available (Table 2, Chitarra, Balestrini, et al., 2014; Koiv et al., 2019; Kutter et al., 2006; Shenoy et al., 2017). Detection of Lm in major plant tissues including vasculature supports its possible transport and dissemination within the plant (Shenoy et al., 2017). Fluorescence in situ hybridization with Lm‐specific oligonucleotides and confocal imaging coupled with immunocytochemistry of a Green Fluorescence Protein‐expressing Lm strain provided evidence of the presence of Lm in plant organs or intercellular spaces of *A. thaliana* leaves (Milillo et al., 2008), carrot, parsley, and celery (Kljujev et al., 2018). The occurrence of Lm in both extracellular and intracellular spaces of lettuce (Shenoy et al., 2017) and sweet corn (Kljujev et al., 2018) was also reported. Surface disinfection followed by enumeration confirmed the endophytic localization of Lm in lettuce and other plants (Chitarra, Decastelli, et al., 2014; Koseki, Mizuno, Yamamoto, 2011; Standing et al., 2013). However, no internalization of Lm was evidenced in other plant species such as barley and basil (Table 2, Chitarra, Decastelli, et al., 2014; Jablasone et al., 2005; Kutter et al., 2006). These plant species‐dependent differences in endophytic colonization by Lm could be linked to the presence or absence of plant metabolites that can either favor or prevent Lm growth. The production of antimicrobial compounds such as essential oils was proposed to limit the colonization of basil by human pathogens (Dorman & Deans, 2000). In summary, conflicting data on Lm internalization requires further comprehensive investigations taking into account factors such as the concentration of inoculum, the method used to detect internalization, the plant genotype/species, which are all known to affect interactions with human pathogenic bacteria (Hirneisen et al., 2012).

## 4 WHY IS THERE SO LITTLE INFORMATION ON PLANT/Lm INTERACTIONS IN LIGHT OF IMMUNITY?

Evolution has shaped defense mechanisms enabling plants to limit the growth of invading microorganisms. The plant immune system relies on the recognition of specific patterns (called Microbe‐Associated Molecular Patterns, MAMPs) on the surface of microorganisms (Jones & Dangl, 2006). Detection of these patterns by pattern recognition receptors (PRRs) localized on the plasma membrane triggers the onset of signaling cascades including a rapid efflux of Ca^2+^, the activation of mitogen‐activated protein (MAP) kinases, and the generation of ROS leading to Pattern Triggered Immunity (PTI) (Pitzschke et al., 2009).

### 4.1 MAMPs and plant immunity

The 22‐amino‐acid flagellin epitope flg22 is one of the most studied MAMPs. It triggers plant responses such as hypersensitive cell death in *A. thaliana* through the binding to the PRR FLAGELLIN SENSING2 (FLS2) (Gomez‐Gomez & Boller, 2000). The second epitope of flagellin, flgII‐28, is sufficient to trigger immunity in *Solanaceae* (Clarke et al., 2013). Flagellin proteins from different bacterial species, pathovars, and strains can display variations in amino acid sequences, and studies have suggested that some phytopathogens can modify their MAMPs to avoid inducing PTI. For example, a single amino acid change in flg22 is sufficient to attenuate or even to block its interaction with FLS2 (W. Sun et al., 2006), and posttranslational modifications of flagellin, including glycosylation, can counteract elicitation (Rossez et al., 2015). Interestingly, MAMPs from commensal, beneficial microbes, and zoonotic human pathogens can be detected by PRRs. As reviewed by Trdá et al., the flagellin and flg22 of the plant growth‐promoting rhizobacteria *P. fluorescens* (WCS374 and WCS417) and the endophytic *Burkholderia phytofirmans* induce an innate immune response in plant cells (Trda et al., 2015). Strategies to evade or suppress plant immunity such as MAMP divergence by sequence variation, MAMP degradation, sequestration, or MAMP modification seem to be similar among commensal, beneficial, and pathogenic microorganisms (Teixeira et al., 2019). Additional MAMPs include elongation factor Tu (EF‐Tu), cold shock proteins, peptidoglycans, and lipopolysaccharides from bacteria, glucans, arachidonic acid, and ergosterol from oomycetes, and chitin from fungi (Boller & Felix, 2009). Interestingly, EF‐Tu, one of the most abundant proteins found in bacteria, triggers an immune response in mammals as well as in plants where PRRs specific to EF‐Tu have been characterized in monocots and dicots (Zipfel et al., 2006). Interaction of PRRs with EF‐Tu involves specific amino acid patterns and is plant‐dependent. The amino acid pattern EFa50 (position 175–225 of EF‐Tu) of *Acidovorax avenae* is recognized by rice PRRs (Furukawa et al., 2014), whereas *A. thaliana* recognizes the pattern composed of the first 18 aa (Kunze et al., 2004).

### 4.2 A contribution of Lm flagellin and EF‐Tu to plant immunity?

**Figure 3:**
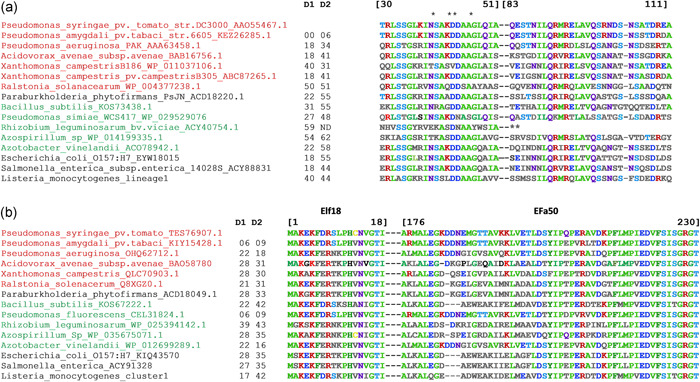
The amino acid sequence of (a) epitopes flg22 and Fl‐II‐28 of Fla and (b) elf18 and EFa50 of EF‐Tu in a selection of bacterial species. *Listeria* sp. sequences of FlaA and EF‐Tu proteins were compared with plant and human bacteria. *Key amino acids of flg22‐eliciting activity in tomato cells (Felix et al., 1999). **No significant homology with fl‐II‐28 of *Rhizobium leguminosarum*. D1 and D2 are sequence divergences (%) calculated with respect to *Pseudomonas syringae*. Plant phytopathogenic bacteria are indicated in red and plant beneficial bacteria in green. Nucleotides are numbered according to the *P. syringae* sequence. Sequence alignment and estimation of sequence divergence (p distance) were performed using MegaX (Kumar et al., 2018)

Perception of zoonotic human pathogens by plants is supported by several studies on *Salmonella enterica* and *Escherichia coli* O157:H7 (Schikora et al., 2008; Teplitski et al., 2012). Indeed the flg22 epitope of these bacteria appears to be perceived by plants and leads to growth restriction of these human pathogens. For example, flg22_St_ of *S. enterica* was found to be an effective MAMP triggering PTI (Garcia et al., 2014), and higher colonization of *A. thaliana* was observed with the flagellum‐defective mutants of *S. enterica* and *E. coli* O157:H7 than with their isogenic parental strain (Melotto et al., 2014). In the case of Lm, however, experimental evidence of a plant immune response triggered by this bacterium is lacking. Therefore we analyzed in silico the available sequences of *flaA* and *tuf*, the *Listeria* genes encoding respectively flagellin and EF‐Tu. The two plant immunogenic epitopes flg22 and fl‐II‐28 are present in the flagellin of Lm and *Listeria* sp. (Figure 3a). No amino‐acid sequence divergence of FlaA was found between *Listeria* species and isolates. At the nucleotide level, the limited divergence between Lm lineages is observed in the sequence of *flaA* (2.3% in total; 1.4% if only the flg22 epitope is considered).

**Figure 4:**
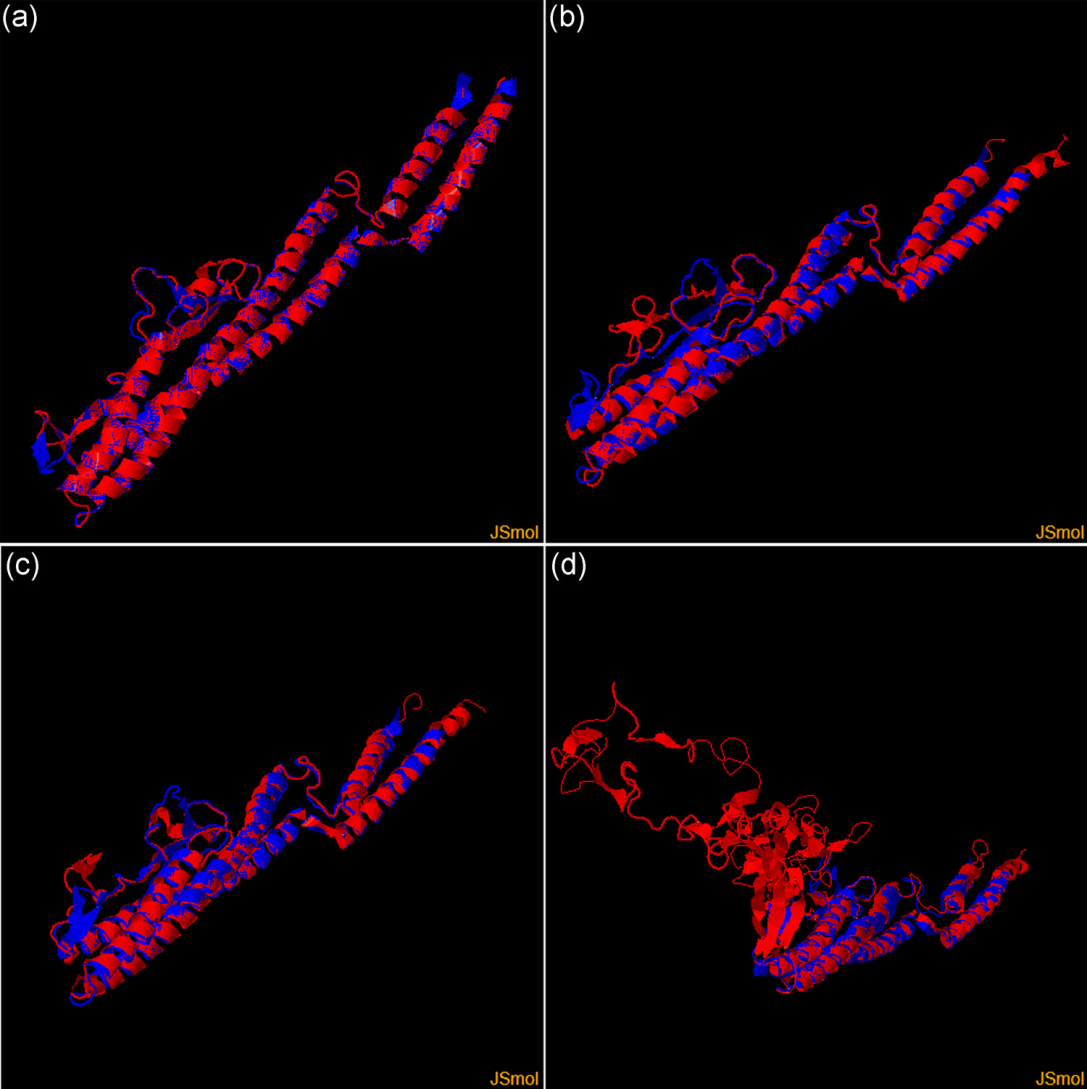
3D model (in red) of flagellin of (a) *Listeria monocytogenes*, (b) *Pseudomonas syringae*, (c) *Bacillus subtilis*, (d) *Azotobacter vinelandii*. The protein structure was predicted by structure homology using the Swiss‐Model utility on the ExPasy server [1] (available online at https://swissmodel.expasy.org/). The four models were built on the top‐ranking template predicted by the software. For comparison, the structures were aligned on a reference (PDB accession: 6PWB.2, in blue) using the TM‐align online tool [2] (available at https://zhanglab.ccmb.med.umich.edu/TM-align/). The model presented a Global Model Quality Estimate (GMQE) of 0.72 for *L. monocytogenes*, 0.91 for *B. subtilis*, 0.74 for *P. syringae*, and 0.53 for *A. vinelandii*

The 3D structure of the flagellin was reconstructed in silico to compare flagellins of Lm, *Bacillus subtilis* (accession number: AOR99902.1), *Pseudomonas syringae* pv. tomato str. DC3000 (accession number AAO55467.1) and *Azotobacter vinelandii* DJ (accession number ACO78942.1). The predicted protein structure shows stable secondary and tertiary structures and suggests conserved conformations in all species (Figure 4). Significant differences were observed within the variable region spanning Gln‐130 to Asn‐185, as expected (Nempont et al., 2008). Interestingly the location of flg22 within a conserved domain at 30–51 aa is common to the four models but variations are observed in the regions surrounding this MAMP. Further biochemical characterization of the flagellin of Lm is required to properly assess protein/protein interactions with the plant receptor FLS2 and the subsequent induction of PTI.

The two plant immunogenic epitopes elf18 and EFa50 of EF‐tu are also conserved in Lm (Figure 3b). They display 83% and 58% identity with the respective sequences from *P. syringae*. At the DNA level, *tuf* nucleotide divergence between Lm lineages is 2.9% (0% and 0.1% for the two EF‐Tu epitopes, respectively). Although this in silico analysis suggests that Lm could trigger PTI after the interaction of these MAMPs with their cognate plant receptors, this has yet to be demonstrated experimentally.

Preliminary experiments in our laboratory failed to evidence plant response to Lm flagellin. Growth of Lm EGDe on seedlings of the Col0 genotype of *A. thaliana* was not modified either in a *flaA* deletion mutant or in a mutant constitutively expressing flagellin (Figure 2a), in contrast to what was reported with other human pathogens such as *S. enterica* or *E. coli* O157:H7 (Melotto et al., 2014). Likewise, impairment of FLS2 did not improve Lm proliferation on *A. thaliana* roots (H.‐N. Truong et al., personal communication). Induction of defense genes or activation of components of the plant's immune response such as MAP kinases could not be evidenced even when very high concentrations of bacteria (10^8^ CFU/plant) were inoculated on roots of *A. thaliana* or *M. truncatula* (Figure 2b). The failure to detect a clear response of plants to Lm could explain the lack of relevant literature addressing the effect of Lm on plant immune response. Further experiments must be designed in which plant/Lm interactions will be investigated in a Systems Biology approach to assess the impact of Lm on plant immunity.

## 5 CONCLUSIONS

Lm is found in a wide range of outdoor habitats though in general at low numbers. In these habitats, including plants, it is usually assumed that Lm can persist as a saprophyte. Plants can indeed provide nutrients readily metabolized by Lm. Reports on preharvest contamination of a variety of crops and vegetables as well as experimental data from plant inoculation with Lm clearly show that plants offer suitable niches for Lm. They can therefore be considered as possible reservoirs of Lm and more generally as reservoirs of human pathogens. From an epidemiological point of view, largescale surveys of preharvest contamination are required to study the intraspecific diversity of Lm isolated from plants. This could help assess whether plant isolates cluster with other environmental and/or clinical isolates or whether specific genomic signatures can be found. It will further document plants as reservoirs of foodborne pathogens potentially leading to contamination of vegetables and fresh produce at retail.

Surprisingly, while reports on intrinsic and extrinsic factors that shape the extent of niches associated with plants are abundant, very few studies focus on their impact on the development of human pathogens, even more strikingly in the case of *Listeria*. Information on the fundamental niche can be retrieved from studies relying on simplified setups of plants grown aseptically, but abiotic and biotic environmental factors narrow down the width of the niches available for Lm on plants. Similarly, the contribution of Lm intraspecific diversity has yet to be considered. Future work will have to address these intrinsic and extrinsic factors to document the realized niche of Lm on plants.

The extent of the interplay between Lm and plants has yet to be clarified. Its intracellular location remains controversial, and whether Lm merely colonizes plant surfaces externally or readily proliferates inside plant cells needs to be fully addressed. No defense response has been observed so far in plants inoculated with this human pathogen although MAMPs (flg22 of flagellin and Ef‐tu) are highly conserved within the species Lm. Further experiments must be designed to determine whether the presence of Lm can trigger plant immune response or conversely if the immune response could be counteracted by the activation of specific bacterial mechanisms upon arrival of Lm on the plant surface.

In conclusion, the data available so far on Lm interacting with plants favor the hypothesis that it can utilize plant‐derived resources to multiply and colonize plant surfaces as a commensal microorganism if competition and antibiosis interactions with the microbiome are permissive enough. A comprehensive Systems Biology approach is necessary to decipher the intertwined interactions between the plant, the microbiome, the pathogen, and the abiotic environment. Association of metabolomics with dual RNA‐Seq approaches and in situ microscopic observations will open a promising avenue of research aiming to characterize the Lm realized niche. Functional genetics approaches could then confirm the role of candidate genes/metabolic pathways in the interplay between the plant, its microbiome, and the pathogen. The triptych microbiome/plant/Lm deserves to be studied as a focal point to keep on improving our understanding of the natural history of this human pathogen.

## CONFLICT OF INTERESTS

None declared.

## ETHICS STATEMENT

None required.

## AUTHOR CONTRIBUTIONS

**Hoai‐Nam Truong**: conceptualization (equal); investigation (lead); project administration (lead); supervision (equal); writing original draft (equal); writing review & editing (equal). **Dominique Garmyn**: investigation (equal); supervision (equal); writing original draft (equal); writing review & editing (equal). **Laurent Gal**: conceptualization (equal); investigation (equal); supervision (equal); writing original draft (equal); writing review & editing (equal). **Carine Fournier**: investigation (supporting); methodology (supporting); visualization (supporting). **Yann Sevellec**: resources (equal); visualization (equal); writing original draft (equal). **Sylvain Jeandroz**: conceptualization (equal); investigation (supporting); writing original draft (equal). **Pascal Piveteau**: conceptualization (equal); project administration (lead); writing original draft (equal); writing review & editing (lead).

## Data Availability

All data generated or analyzed during this study are included in this published article.
